# Radar-Based Contactless Monitoring in Different Inpatient Nursing Care Settings: Mixed Methods Feasibility Study

**DOI:** 10.2196/93624

**Published:** 2026-08-28

**Authors:** Madeleine Ritter-Herschbach, Sebastian Hofstetter, Dominik Behr, Melina Hager, Burkhard Mueller, Patrick Jahn

**Affiliations:** 1 Health Service Research Working Group, Faculty of Medicine, Martin Luther University Halle-Wittenberg, Ernst-Grube-Str. 40, Halle, Saxony-Anhalt, 06120, Germany, 49 03455571348; 2 Dorothea-Erxleben-Lernzentrum, Faculty of Medicine, Martin Luther University Halle-Wittenberg, Halle, Saxony-Anhalt, Germany; 3 University Clinic and Polyclinic for Geriatric Medicine, University Hospital in Halle, Halle, Saxony-Anhalt, Germany; 4 University Clinic and Polyclinic for Internal Medicine II, University Hospital in Halle, Halle, Saxony-Anhalt, Germany

**Keywords:** vital signs monitoring, radar-based sensors, patient safety, inpatient care, nursing care process, usability

## Abstract

**Background:**

Monitoring of vital signs is a critical component of nursing care, facilitating the early detection of complications, enabling the observation of the progression of health status, and providing a foundation for diagnosis and therapeutic decision-making. It is instrumental in ensuring quality of care. Contactless measurement methods, such as radar-based sensors, have the potential to enable continuous monitoring of respiration, heart rate, and bed activity without disturbing or stressing vulnerable patients.

**Objective:**

The study investigates the feasibility, usability, acceptance, and implementation of a contactless digital vital-signs monitoring system. The objective is to ascertain the suitability of the device for incorporation into the workflow of nursing personnel and to assess implementation factors in an inpatient setting. Additionally, the study examines user acceptance and satisfaction among nursing staff.

**Methods:**

A total of 13 nursing staff members from geriatric and dialysis departments participated in this mixed methods pilot study. Following the installation of the technology, the participants underwent a 30-minute training period to become acquainted with its operation. Thereafter, the system was subjected to a 4-week trial period. Quantitative data were collected at 3 points in time using the Technology Usage Inventory and the System Usability Scale. Furthermore, measurement protocols were evaluated to compare the time required for the standard and contactless measurement methods. Qualitative data were collected subsequent to the conclusion of the trial through the implementation of focus group interviews. Ethical approval and informed consent were obtained.

**Results:**

The study results suggest that the average intention to use among the study participants (n=13) reached a relatively high level after 4 weeks (mean 85.5, SD 107.7). However, variation was observed when the 2 nursing wards were considered separately (geriatrics: 28.4 vs dialysis 199.8). The monitoring system was evaluated based on the System Usability Scale, which yielded a mean score of 74.5 (SD 10.5), indicating good usability. The nurses reported a perceived enhancement in their sense of security and monitoring oversight during routine care. Additionally, potential time savings were observed due to a reduction in disruptions to established processes. However, compared with conventional real-time monitoring, disadvantages were identified and technical requirements were defined.

**Conclusions:**

The use of contactless measurement in the assessment of vital signs holds considerable promise for enhancing the efficacy of nursing care, particularly in contexts where manual measurements have been the prevailing standard. In order to further explore technical capabilities in a nursing context, additional research needs to be conducted.

## Introduction

### Contactless Monitoring Systems in Hospital Care

The continuous monitoring of vital signs constitutes a fundamental element of professional nursing care and clinical decision-making. The regular assessment of parameters such as heart rate, respiratory rate, and physical activity enables early identification of clinical deterioration, supports timely interventions, and contributes to patient safety [1]. Beyond its clinical relevance, systematic monitoring and documentation of vital signs also play a crucial role in quality assurance processes and provide legal protection for nursing staff by ensuring transparency and traceability of care interventions.

In many inpatient settings, vital signs are still manually measured at predetermined intervals [2,3]. Conventional monitoring methods often require direct physical contact with patients, which can impose a significant burden or elicit feelings of stress. Furthermore, this contact has been identified as a risk factor for the development of delirium [4], particularly among vulnerable populations, including older patients, individuals with cognitive impairment [5], patients who had a stroke [6], and those with fragile skin conditions [7]. Repeated manual measurements can disrupt patients’ rest and sleep. This disruption may, in turn, have a deleterious effect on the recovery and overall well-being of the individual.

Advancements in digital health technologies have precipitated the development of contactless monitoring systems, including radar-based sensors capable of detecting micromovements of the chest wall [8]. These systems provide continuous monitoring of respiration, heart rate, and bed activity without the use of electrodes, cuffs, or wearable devices. In addition to enhancing patient comfort, contactless monitoring technologies have the potential to reduce nursing workload, support early detection of critical events, and enhance safety within care processes. However, the extent of evidence regarding the usability, acceptance, and integration of these tools into nursing workflows remains limited [9].

### Aim

The present study explores the feasibility and usability of implementing a contactless digital monitoring system (Neteera 130H-Plus) [10] within a hospital environment. The aim of this study is to determine the extent to which the device integrates into the workflow of nursing personnel, to assess the device’s acceptance among users, and to identify potential barriers to implementation. From the perspective of the users, the following assessments will be formulated: time savings, work simplification, satisfaction, and usability in everyday clinical practice.

## Methods

### Study Design

This study was designed as a feasibility study to assess the usability and feasibility using a mixed methods approach [11,12]. The study used a sequential explanatory design, combining quantitative and qualitative data collected in 2 consecutive phases. The qualitative phase followed the quantitative phase to further explain and contextualize the results. This approach was selected to comprehensively evaluate the feasibility, usability, and acceptance of the Neteera 130H-Plus system in routine clinical nursing care. Methodological reporting was carried out using the GRAMMS (Good Reporting of a Mixed Methods Study) checklist [13] (Checklist 1).

**Figure 1.**
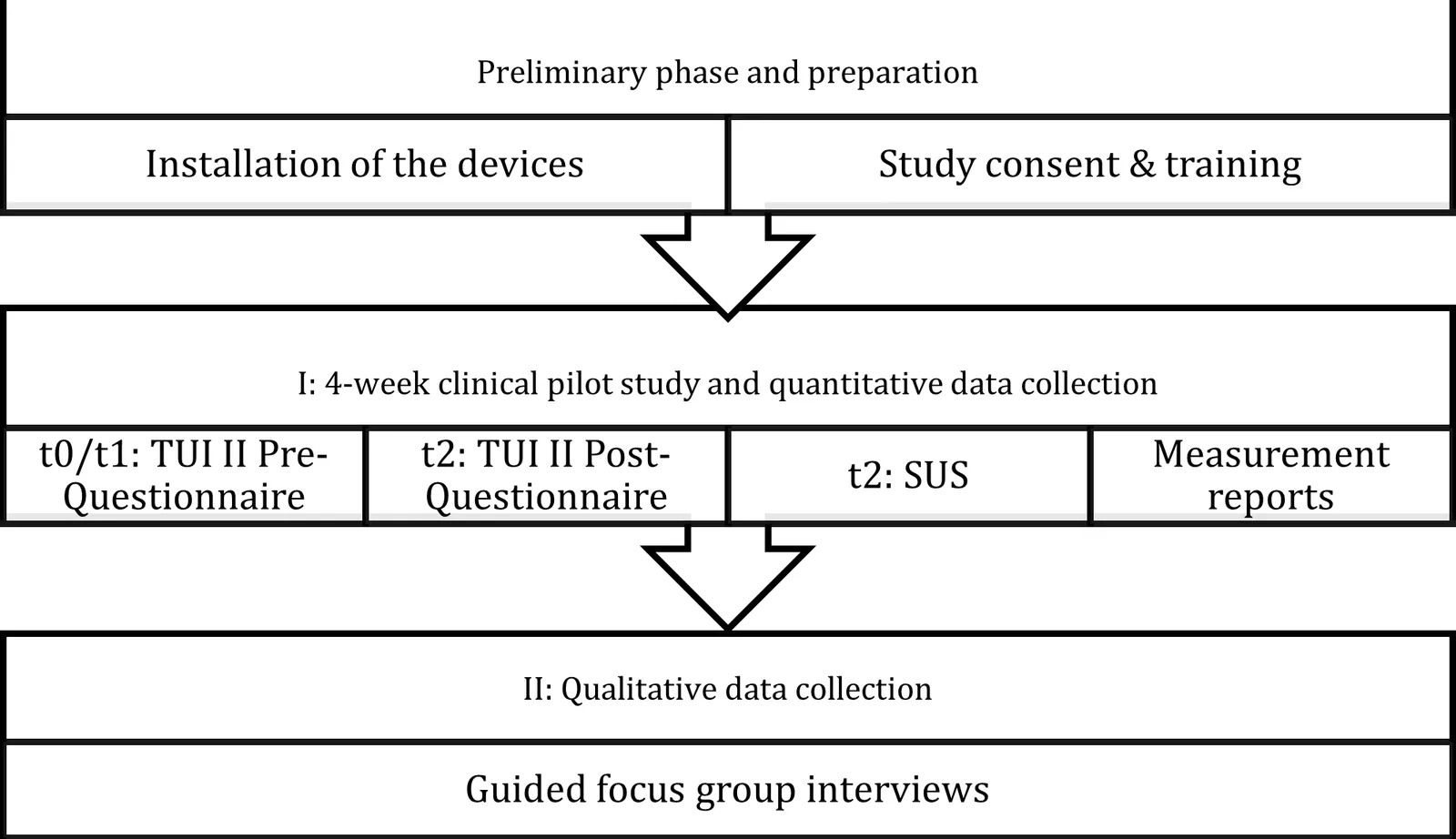
Study design and procedure. SUS: System Usability Scale; TUI: Technology Usage Inventory.

The study was conducted under real-world conditions without altering standard clinical workflows. Patients were monitored using both the Neteera system and standard-of-care monitoring devices, enabling direct comparison without influencing clinical decision-making. Following a preparatory phase that included device installation, consent procedures, and staff training, a 4-week clinical pilot phase was conducted with quantitative data collection. The Technology Usage Inventory II (TUI II) [14] was used to assess acceptance at baseline (T0/T1) and postintervention (T2). System usability was measured using the System Usability Scale (SUS) [15] at T2. Concurrently, measurement reports and time records were generated. Subsequently, qualitative data were gathered through guided focus group interviews to contextualize and expand upon the quantitative findings (Figure 1).

In this study, *usability* refers to the ease of use of the system, *feasibility* to its practical implementation under routine clinical conditions, *usefulness* to the perceived benefit to nursing work, *acceptance* to the overall evaluation of the technology, and *intention to use* to the willingness to continue using the system in clinical practice.

### Setting and Participants

The study was conducted at a German university hospital in 2 inpatient clinical departments: an acute dialysis department and a geriatric ward. The wards were selected to reflect different clinical settings, each with distinct monitoring requirements and workflows. The study population comprised registered nurses and nursing professionals involved in the routine monitoring of vital signs in inpatient, predominantly bedbound patients. The inclusion criteria comprised active involvement in vital sign monitoring and direct use of the Neteera system during clinical shifts. Participation in the study was voluntary and conducted during regular working hours. The study sample included all nursing staff available at the time of recruitment in the 2 wards. The sample size was selected pragmatically, in line with the exploratory nature of a feasibility study and without a formal power calculation. Patients were not actively recruited for study participation; however, they were provided with study-specific information about the technology being tested. Monitoring was performed exclusively in the context of routine clinical care. All patients continued to receive standard monitoring, independent of the study, ensuring that no clinical decisions were based on data generated by the investigational system.

### Intervention

**Figure 2.**
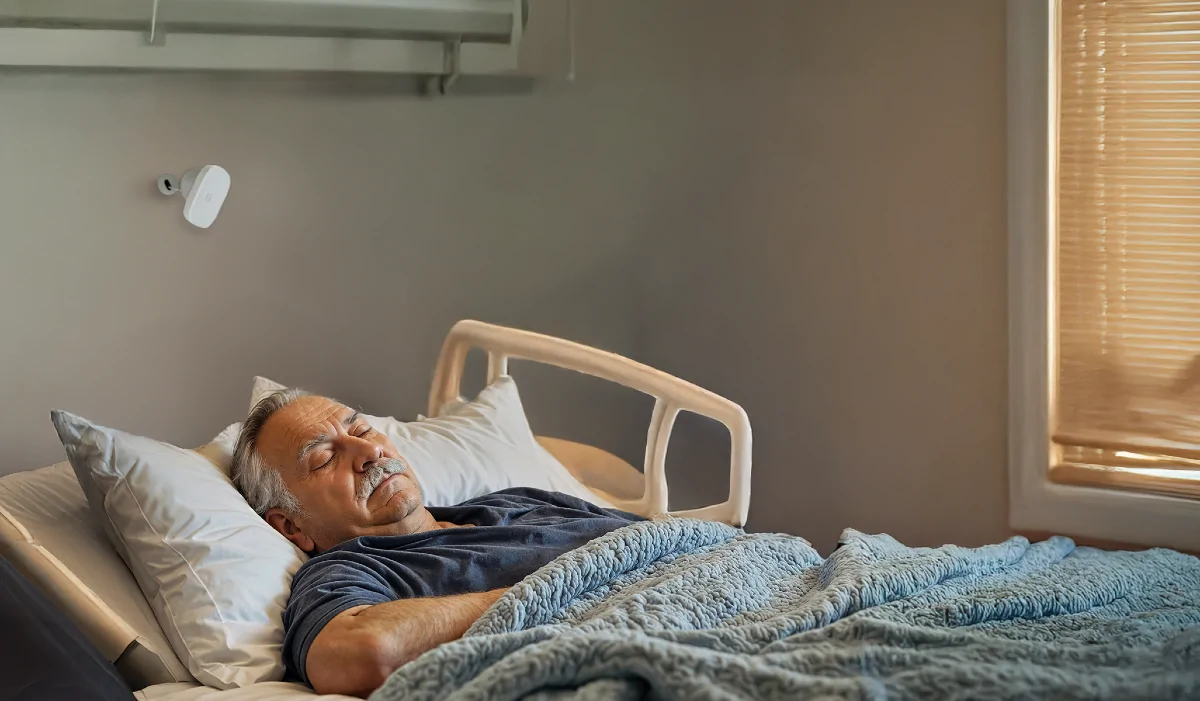
Central positioning of the “Neteera” sensor (Neteera product photo).

The Neteera 130H-Plus is a contactless, radar-based remote patient monitoring system designed to continuously capture health parameters (heart rate, heart rate variability, respiration rate, depth, and amplitude) and activity tracking (micromovements, time in bed, and bed exits) without direct patient contact. The device is mounted above the patient’s bed (on the wall or headboard) and operates through clothing and bedding (Figure 2). All data are transmitted in encrypted form to a cloud-based platform hosted on European Union–based servers. The nursing staff underwent a structured, 30-minute training and onboarding session prior to system implementation. A live demonstration of the available functions was provided at the nursing station, followed by immediate system testing. The research staff were on site at all times to answer any questions.

### Quantitative Data Collection and Analysis

#### Acceptance, Usefulness, and Usability

The TUI II [14] was used to assess technology acceptance and perceived usefulness at 3 distinct time points: baseline (T0, following the training), mid-study (T1, after 2 weeks of use), and end of study (T2, after 4 weeks of use). The TUI methodically assesses numerous dimensions. The TUI II is a parallel version of the TUI and is used to minimize learning effects or measurement repeatability errors when participants complete the questionnaire multiple times (eg, for preinteraction and postinteraction comparisons with a technology). At T0, the “TUI-II Pre” was administered. The questionnaire comprises 8 items, which relate to both the new technology and the general attitude toward technologies, and it allows statements to be made on the “curiosity” and “technology anxiety” scales. At T1 and T2, the “TUI-II Post” was used to assess 6 scales: “interest,” “accessibility,” “usefulness,” “ease of using a technology,” “skepticism,” and “intention to use (ITU),” comprising a total of 18 items [14]. The ITU scale is a 10-centimeter-long visual analog scale used to assess agreement with given statements. The end points of the scale (eg, “agree” vs “disagree”) serve as anchors for assessing the degree of agreement. The distance between the scale end point representing complete disagreement and the participant’s mark is measured in millimeters for each of the 3 items. The 3 values are then summed to obtain an ITU score ranging from 0 to 300. The ITU scale assessed the ITU, intention to purchase, and desire for access to the technology. Lower scores indicate higher ITU, willingness to purchase, and readiness for access. Usability was additionally assessed using the SUS [15]. The SUS was administered after 4 weeks of system use (T2) and consisted of 10 items rated on a Likert scale.

#### Time Required for Vital Sign Measurement

The potential time savings were assessed by documenting the time required (in seconds) to monitor pulse and respiratory rate, both with and without the Neteera device. The documentation was carried out by a nurse in the geriatric ward, who followed a standard protocol. The following components were documented: preparation, measurement, documentation, and deviations or delays during measurement.

The quantitative data were analyzed descriptively using IBM SPSS Statistics version 2. The results were reported as mean scores and SDs according to the respective scoring manuals.

### Qualitative Data Collection and Analysis

Following the testing phase and completion of the quantitative surveys, focus group interviews were conducted with nursing staff from both wards to identify experiences, perceived benefits, barriers, and implementation requirements. The focus groups were conducted using a semistructured interview guide. The guide was developed in accordance with established standards of qualitative research and tailored to the research objectives (see Multimedia Appendix 1). The interview guide explored participants’ initial impressions and practical experiences with the Neteera system, with a particular focus on usability and its integration into daily nursing workflows. The investigation further examined the perceived benefits and limitations regarding patient safety, quality of care, and the influence of specific clinical settings on system use. Finally, participants were asked to reflect on future roles, necessary improvements, and overall expectations for the implementation of contactless monitoring technologies in nursing practice.

All discussions were audio-recorded, transcribed verbatim, and pseudonymized prior to analysis. Qualitative content analysis was conducted in accordance with the structured approach proposed by Kuckartz and Rädiker [16] using the MAXQDA 2022 software (VERBI Software GmbH), integrating deductive and inductive category development. This method is particularly well-suited for the systematic analysis of extensive qualitative data. It integrates deductive category formation based on the research questions with inductive further development from the material. The transcripts were initially coded according to the interview guidelines and research objectives by one researcher and subsequently by a second researcher. Subsequently, the discrepancies in the coding were reviewed and discussed. Additional subcategories were inductively added based on the material. The transcripts were coded thematically to identify recurring patterns, perceived benefits, and challenges related to system use and integration into nursing care processes.

The qualitative findings were used to explain and contextualize the quantitative results, particularly differences in usability, acceptance, and perceived usefulness across the 2 clinical settings.

### Ethical Considerations

The study was conducted in accordance with the Declaration of Helsinki and Good Clinical Practice guidelines. Ethical approval was obtained from the responsible institutional ethics committee prior to study initiation. All participating nursing professionals provided written informed consent. Data were pseudonymized and stored in encrypted form in accordance with the General Data Protection Regulation. The study received ethics approval from the ethics committee of the Medical Faculty of Martin Luther University Halle-Wittenberg (approval number 2025‐047, April 30, 2025) and was registered at the German Clinical Trials Register (DRKS00036910). Written informed consent was obtained from all participants, and no financial compensation was provided.

## Results

### Pilot Study Outcomes

A total of 16 devices were installed in the 2 wards, distributed across 7 patient rooms (n=3 geriatrics, n=4 dialysis). The study staff administered 3 training sessions on site, each lasting approximately 30 minutes. A total of 21 nurses were trained in the system and its use (n=14 geriatrics, n=7 dialysis). The 4-week clinical trial phase began immediately afterward.

### Quantitative Results

In the context of geriatric care, a total of 9 (64.3%) nurses completed the questionnaire survey. In the dialysis care setting, 4 (57.1%) nurses completed the survey.

#### Technology Acceptance

High scores on the TUI II scale indicate a high degree of expression within the designated dimension. Conversely, low scores indicate a low degree of expression [14]. Table 1 presents the mean values obtained from the scales at the 3 distinct measurement time points. For scales with 3 items, the total scale value ranges between 3 and 21 (b), and for scales with 4 items, between 4 and 28 (a). The highest values were recorded on the scales “interest” (T1=22.1; T2=20.9) and “usefulness” (T1=22.6; T2=23.6), with the former decreasing as expected and perceived usefulness being highest at the end of the trial. Skepticism decreased over the 4-week observation period (T1=8.3; T2=7.1), although it was already very low at the baseline. Due to missing responses, not all scales could be calculated in full. Consequently, the total sample size varies and is reported separately for each measurement point in column N.

**Table 1.** Technology Usage Inventory II subscale scores across measurement time points (T0-T2).

Scale	Range	T0		T1		T2	
		n	Mean (SD)	n	Mean (SD)	n	Mean (SD)
Curiosity	4‐28	15	17.6 (5.1)	—^a^	—	—	—
Fear of using technology	4‐28	16	8.0 (3.2)	—	—	—	—
Interest	4‐28	—	—	14	22.1 (3.9)	15	20.9 (3.7)
User-friendliness	3‐21	—	—	11	18.9 (1.8)	13	18.2 (2.3)
Usefulness	4‐28	—	—	14	22.6 (5.5)	14	23.6 (3.2)
Skepticism	4‐28	—	—	10	8.3 (3.6)	10	7.1 (3.0)
Accessibility	3‐21	—	—	5	12.0 (3.4)	6	11.8 (3.6)

a Not applicable.

The ITU subscale was also examined using a group-specific evaluation (geriatric ward and dialysis) of the 3 subscales. This finding explains the substantial disparities observed, the origin of which warrants further investigation through a subsequent qualitative survey of nursing personnel. The maximum total score that can be attained is 300, with higher scores indicating a lower willingness to use. As demonstrated in Table 2, a clear discrepancy in scores is observed between the 2 wards. The geriatric ward demonstrated low total scores of 40.8 (T1) and 28.4 (T2) on the ITU scale, indicating a high willingness to use. In contrast, the dialysis respondents showed a heightened degree of caution, as evidenced by a diminished propensity to use the device with scores of 195.5 (T1) and 199.8 (T2).

**Table 2.** Intention-to-use (TUI^a^ II) scores in geriatric and dialysis wards at T1 and T2^b^.

TUI II item	T1, mean (SD)		T2, mean (SD)	
	Geriatrics	Dialysis	Geriatrics	Dialysis
Would you want to use this technology?	12.6 (16.0)	66.3 (37.3)	12.6 (16.0)	12.6 (16.0)
Would you purchase this technology?	19.7 (28.4)	66.8 (37.8)	16.2 (24.9)	65.0 (35.3)
Would you want to have access to this technology?	8.6 (10.1)	62.5 (34.4)	5.1 (8.1)	66.2 (37.4)
Total intention to use	40.8 (51.2)	195.5 (109.0)	28.4 (41.8)	199.8 (111.3)

a TUI: Technology Usage Inventory.

b Lower values indicate a higher intention to use.

#### System Usability

The mean overall SUS score was 74.5 (SD 10.5), with values ranging from 58 to 98. According to the SUS manual, a system is considered usable if it attains a score of 68, which was achieved in this study. A comparison of ward-specific scores showed that the SUS score was 76.2 (SD 12.1) for the geriatric ward and 71.0 (SD 5.7) for the dialysis ward. This indicates slightly lower perceived usability in the dialysis ward compared with the geriatric ward. The nursing staff (n=15) evaluated a series of statements using a scale ranging from 1 (“strongly disagree”) to 5 (“strongly agree”). These are presented in Multimedia Appendix 2. The system is described as straightforward to operate (ie, ease of use), with minimal training requirements. The values align with the ITU results on the TUI II scale.

#### Time Saving

A total of 31 measurement situations were meticulously observed and documented in the geriatric ward using a standard measurement protocol. The vital parameters of heart rate and respiratory rate were determined through the measurement of time in seconds. As shown in Table 3, radar-based measurement was considerably faster, with an average completion time of 20.3 (SD 16.2) seconds compared with 133.8 (SD 69.8) seconds for the conventional method. In 7 (22.6%) cases, delays or challenges in using the Neteera system were described. In 5 instances, the values were not displayed in real time at the desired moment. In 2 cases, it was necessary to reenter the user credentials due to a system logout that occurred in the interim.

**Table 3.** Comparison of the standard measurement method and the Neteera system (in seconds) (N=31).

Measurement method	Mean (SD, range) (s)
Manual measurement	133.8 (69.8, 50-380)
Radar-based measurement	20.3 (16.2, 6-60)

### Qualitative Results

#### Findings from the Focus Groups

Following the completion of the trial period, 2 focus groups were conducted, comprising 4 participants in the geriatric ward and 3 in the dialysis unit. The interviews lasted between 26 and 32 minutes. Initially, primary categories were delineated in accordance with the research interests. In an iterative process, these categories were refined during the analysis and supplemented with subcategories. The coding of the 8 primary categories was conducted in close proximity to the data and was grounded in the perspectives of the nurses interviewed. The coded text passages were subsequently summarized by category and condensed for content.

#### Handling and Usability

The category of handling and usability includes statements on the user-friendliness of the dashboard, training, and practical limitations in everyday use. The operation of the system was characterized as uncomplicated and intuitive. Even those with limited technological expertise reported that the system was straightforward to use. The training was deemed adequate:

*Well, I have to say, I'm from a generation that didn't grow up with computers and I don't know a whole lot about them, but I got along just fine. I think it’s easy to use. I think we can all manage it*.

However, it was noted that external factors, including bed position, sensor placement, or interference (eg, bed gallows, elevated positioning), can substantially impact transmission stability. Repeated display failures resulted in additional work and frustration, as the nursing staff were compelled to examine the cause of the missing measurements directly at the patient’s bedside. These findings are consistent with the quantitative usability results, as reflected in the overall SUS score of 74.5, indicating good usability and ease of use.

#### Integration Into Nursing Workflows

Integration of the system into existing workflows emerged as a pivotal category. The ability to collect data without directly disturbing patients, especially during nocturnal hours, was highlighted as a positive feature. Caregivers reported that nighttime routine activities, such as medication preparation, were interrupted less frequently and that there was a greater sense of security because vital signs could be monitored continuously:

*It simply means that we can collect data at night without having to wake patients*.

The system was perceived as beneficial due to its capacity to offer an overview of patients’ conditions, thereby obviating the necessity for repeated in-person visits. From the participants’ perspective, these measures contributed to enhanced work organization and a subjective sense of safety.

This perceived advancement in workflow integration is consistent with the high ratings achieved in the TUI II dimensions of “usefulness” and “user-friendliness” (Table 1).

#### Positive Usage Situations

This category comprises specific clinical application scenarios in which the Neteera system has been demonstrated to be particularly beneficial. The aforementioned participants primarily included medical and nursing issues in which progress data contributed to clinical decision-making.

One example cited was its use in patients with respiratory diseases, where changes in breathing could be monitored over time:

*Well, I once had a patient with COPD who we had newly prescribed a new spray for, and she was always experiencing acute shortness of breath. The doctor came and asked how her breathing was, so we looked at the progression and saw what the curve looked like, and that helped us at that moment. Because we could see in black and white how things had progressed. I think that’s great for things like that.*

The system was also described as helpful in situations requiring continuous monitoring in general wards, as it enabled selective, close observation without requiring continuous physical presence.

The findings from these cases align with the high perceived usefulness reported in the quantitative data (TUI II), particularly in the geriatric ward, where there was a high level of ITU.

#### Negative Usage Situations

Within this category, critical feedback is summarized. A large number of complaints concerned the lack of real-time measurements and unclear update intervals for vital signs. This was perceived as a significant limitation in nursing areas that performed circulation-intensive treatment procedures:

*But we couldn't see on the tablet how often the heart rate was updated. So we couldn't tell, because it wasn't a real-time measurement, it was a fixed number that stayed the same. Our [dialysis] ECG monitoring [electrocardiogram] really shows the current heart rate, and that changes constantly*.

In addition, participants reported frequent measurement failures and significant deviations from established standard procedures (eg, electrocardiogram monitoring). The observed discrepancies have led to a decline in confidence in the accuracy of the measured values. As a result, interest in using the system decreased.

*Everyone stood there comparing their patients, but then something didn't work and interest waned. Yes. You could tell*.

These limitations are reflected in the lower ITU values at the dialysis unit (Table 2). This finding is consistent with the independent benefit because lower ITU values can be interpreted as a prerequisite for subsequent use. The qualitative results provide a valuable explanation for the quantitative differences observed between the units, particularly the lower acceptance levels in contexts requiring real-time monitoring and high measurement reliability.

#### Opportunities and Benefits for the Nursing Process and Outcomes

A wide range of potential benefits of the system for the nursing process was identified. Wireless monitoring and the reduction of physical constraints were perceived as key benefits for patients. These features were expected to enhance safety and reduce agitation, particularly in patients at risk of delirium, restlessness, or falls.

*Actually, all of the patients we have here [geriatric ward] are at risk of delirium. Patients who have delirium definitely benefit from it. Patients who have a disturbed day-night rhythm definitely benefit from it. Hypoactive delirious patients.*

The ability to perpetually assess the respiratory rate, a parameter that has not been systematically documented in conventional nursing practice, has furnished nurses’ novel perspectives into the early identification of stress, anxiety, or pain:

*Without this technology, we would not do this, of course. We would not measure respiratory rate at night. If it can be collected in this way, we can then draw conclusions and take action. Perhaps the patient is in pain, perhaps they are anxious, and so on and so forth*.

Earlier detection of nocturnal events may be possible without interrupting patients’ sleep. These perceived benefits are consistent with the high ratings for “usefulness” and “interest” found in the quantitative results. The findings are complemented by the qualitative data, which illustrate how continuous monitoring can improve patient safety and support clinical decision-making under specific conditions.

#### Nursing Workload and Stress

The system received a mixture of favorable and unfavorable reviews with regard to its workload. In situations involving high levels of stress, the implementation of automated vital sign monitoring has been shown to result in a substantial reduction in workload, primarily by eliminating the need for time-consuming manual measurements. The use of this technology has been demonstrated to reduce the necessity for direct observation, thereby minimizing disruptions and enhancing the perception of security:

*I would have had to check in every five minutes. We were still there, regularly, but still, you felt… you felt more at ease.*

*But for patients at risk of falling who now need some kind of bedside attendance, this could of course lead to a certain reduction in staff numbers in the short term.*

At the same time, participants emphasized that these benefits depend on the system functioning reliably and consistently. These observations are consistent with the time savings identified in the quantitative data, which point to reduced measurement times. The qualitative results indicate that the benefits are contingent on the system’s reliability and on further advancements in its functionality, such as blood pressure measurement.

#### Future Potential and Economic Aspects

The participants formulated explicit expectations for the subsequent evolution of the system. The integration of additional vital signs, especially blood pressure, was identified as an important requirement. The implementation of comprehensive digital documentation, accompanied by a direct link to the hospital information system, would also constitute a considerable development. Furthermore, reliable real-time data transmission and bedside alerting were identified as key requirements for future system development:

*It’s also ideal for some areas of nephrology. It’s particularly ideal for adjusting blood pressure medication. It would be great to be able to see what happens or would happen an hour after administering medication*.

*Well, I think that in order to implement this permanently, you would need improved programme stability so that there are fewer disruptive factors, and a HIS interface.*

The system was perceived as potentially cost-effective, especially compared with conventional wired monitoring systems:

*That’s very affordable, because I recently replaced some cables on a monitor and that cost €400 for just one monitor. That would be quite interesting for us.*

These expectations for further system development are consistent with the high overall interest and willingness to engage with the technology reported in the TUI II results (Tables 1 and 2). The qualitative data suggest that future adoption depends on meeting the identified technical and structural requirements.

#### Challenges and Barriers to Implementation

Finally, technical, organizational, and structural barriers were discussed. While nurses were generally open to digital systems and did not perceive a risk of dehumanized care, they expected some patients to be skeptical of the technology.

*This is also extremely important for nursing staff. It has nothing to do with dehumanization or anything like that. It’s simply about having the security of having data available at night as well*.

A perceived need for additional patient information was identified:

*Many [patients] were initially worried that it was a camera, that they were being filmed. That was always their first thought, but then they were actually quite open. No one said, “No, for God’s sake, I don't want that”*.

The primary barriers to implementation identified included a high susceptibility to malfunction, an absence of real-time capability, inadequate interfaces, and an insufficient trial period. It was also emphasized that the system’s full potential could only be realized if all rooms were fully equipped and seamlessly integrated into existing IT structures.

The identified barriers provide a contextual framework for understanding the variability in quantitative acceptance scores, particularly the lower ITU in more technically demanding settings (Table 2). Technical reliability and system integration emerged as critical factors for successful implementation.

## Discussion

### Principal Findings

The objective of this study was to assess the feasibility and applicability of implementing a contactless, radar-based monitoring system in an inpatient setting from the perspective of nursing professionals.

The findings indicate that the Neteera 130H-Plus system is perceived as usable and potentially beneficial for nursing care, particularly in settings where vital signs are traditionally measured manually and intermittently. While quantitative results support the system’s usability, qualitative data provide additional insights into the integration of nursing workflows, perceived safety gains, and setting-specific limitations. The findings indicated notable discrepancies in acceptance and ITU between the geriatric and dialysis wards. The results underscore the pivotal role of contextual factors in the implementation of digital monitoring technologies within nursing practice.

### Technology Acceptance and ITU

The quantitative results indicate a high level of acceptance of and willingness to use radar-based, contactless monitoring among nursing staff, particularly in the geriatric ward.

The SUS score indicates that the system is usable and exceeds the commonly accepted threshold [15], suggesting that the system can be handled effectively by nursing staff with limited training. Participants described the system as easy to use, suggesting a high level of usability. This finding is consistent with previous studies on digital assistive technologies in inpatient care. The extant literature demonstrates that usability is a prerequisite for sustained adoption in clinical practice [17], but it is not sufficient on its own.

The TUI II subscales “interest” and “usefulness” showed consistently high scores throughout the observation period. However, perceived interest demonstrated a slight decline, while perceived usefulness reached its peak at the conclusion of the 4-week test phase. This finding suggests that the nursing staff regarded the implementation of the system as both practical and conducive to the effectiveness of nursing processes. Concurrently, the degree of skepticism remained minimal, underscoring a fundamental openness to the technology. A thorough analysis of the TUI II subscales reveals variations between the wards. In the geriatric ward, the values were found to be particularly low, which correspond to a high level of willingness to use the system. In contrast, the dialysis ward exhibited elevated values, indicating a more cautious approach among nursing staff. The observed discrepancy can be attributed to context-specific implementation factors. The qualitative findings indicate that acceptance was shaped not only by system usability but also by the degree to which the technology aligned with existing monitoring practices, workflow requirements, and expectations regarding real-time information. Workflow and monitoring requirements were in place due to the standard practice of continuous monitoring and real-time feedback in the dialysis ward (eg, via electrocardiogram and blood pressure measurement). Consequently, nursing staff required immediate access to relevant data that are continuously updated, particularly in situations involving medical decisions and emergency situations. The lack of reliable real-time data and concerns about measurement accuracy diminished the perceived relevance of the data for nursing decision-making. A recent systematic review described deviations in radar-based measurement methods. The review emphasizes that measurement accuracy is highly dependent on the measurement environment and conditions. Interfering factors, such as movement or device positioning, may influence measurement accuracy. The present findings are consistent with the observations made in the study [8].

Moreover, the system enables continuous vital sign monitoring without disturbing patients, a novel feature particularly relevant during nocturnal hours in the geriatric ward. The participants reported that continuous monitoring without disturbing patients contributed to a perceived sense of security among nursing staff. This finding is consistent with previous literature, which demonstrates that contactless monitoring is particularly well-received in settings involving vulnerable patients, as it enhances comfort and safety [18].

### Time Savings and Impact on Workload

During the pilot phase, a key benefit identified was the reduction in time required to measure selected vital signs using radar-based monitoring. However, the potential for greater time savings remains limited in the absence of radar-based blood pressure measurements. The findings should be interpreted with caution, as the calculations were based on data from a single nurse and were not performed under controlled conditions. Further comparative measurements under controlled conditions are needed to draw reliable conclusions. The qualitative findings indicate that a reduction in interruptions, a decline in visits to the bedside, and the implementation of continuous background monitoring contributed to a subjective feeling of relief and enhanced safety, particularly during night shifts. However, nursing staff also emphasized that the efficacy of these benefits is contingent upon the stability of the system. The occurrence of technical issues, including delayed data display and forced logins, reduced the initially observed time savings. These issues also increased workload and frustration among nurses. Consequently, the efficacy of time savings appears to be contingent on the reliability of system performance [19] and the availability of blood pressure measurements.

### Strengths and Limitations

This design enabled the contextualization and interpretation of quantitative usability and acceptance data through qualitative insights. The qualitative findings helped explain why most participants perceived the system as easy to learn and use despite occasional technical difficulties. The integration of 2 distinct clinical wards allowed the exploration of different nursing workflows and monitoring requirements, which is particularly relevant for feasibility research [19]. Conducting the study under real-world conditions without modifying standard care processes strengthened ecological validity and provided realistic insights into everyday nursing practice. It is imperative to acknowledge the active involvement of academically trained nursing staff, particularly those specializing in geriatrics, in the clinical setting. Consequently, while the time expenditure measurements were conducted on a limited scale and are not generalizable, they were executed under real-world conditions. In light of a single observer, one ward, and the limited number of observations, the findings related to time-measurement should be interpreted as exploratory observations rather than estimates of implementation-related efficiency gains.

Several methodological limitations should also be considered. The sample size was limited and based on pragmatic considerations, which is common in feasibility studies but restricts generalizability. Furthermore, the 4-week intervention period may not have been sufficient to establish stable routines or to assess long-term acceptance, particularly in technically demanding clinical environments.

### Implications for Nursing Practice

The observed discrepancies among nursing wards underscore the notion that the efficacy of implementation is contingent upon the prevailing context. In settings characterized by acute demands for real-time data, such as dialysis nursing care, the necessity for robust and frequently updated systems is paramount to ensure trust and use. In less intensively monitored areas, such as geriatrics, the implementation of a system that provides vital parameters continuously, albeit not necessarily in real time, has been demonstrated to effectively alleviate workflow burdens, support monitoring processes, and reduce workflow interruptions from the perspective of nursing staff [20]. From the perspective of the nurses surveyed, blood pressure measurements play a decisive role in the complete mapping of basic parameters. Therefore, the potential time savings in vital sign measurement can only be partially evaluated, as blood pressure and pulse are usually measured in separate cycles. While intuitive design and ease of use are frequently associated with positive user perceptions, broader contextual factors such as workflow integration, organizational support, training, and alignment with clinical needs fundamentally influence whether technologies are sustainably adopted in nursing care environments [17,21].

Despite the system’s capability for activity tracking, this feature had little influence on nurses’ evaluations. The use of activity data for clinical decision-making was limited, as these data were often perceived as less relevant than vital parameters such as respiratory rate and heart rate. In some acute care settings, nursing staff have reported that automated activity tracking is of secondary importance compared with direct clinical observation, which may reduce the added value of such systems in these contexts. The quantitative and qualitative findings suggest that activity tracking may be more relevant in long-term care or settings with lower staff-to-patient ratios, where continuous direct observation is less feasible. In these contexts, changes in movement patterns may serve as early indicators of functional decline, delirium, or fall risk [22]. The limited use of this feature in the present study does not necessarily reflect a lack of potential value but highlights the importance of adapting technological functionalities to the specific needs and constraints of the care environment.

### Implications for Future Research

In order to validate these findings and investigate acceptance and usability across a broader range of clinical contexts, larger, multicenter studies are necessary. A comparative analysis of contactless monitoring systems and established monitoring technologies could provide further insights into the added value of radar-based approaches. The potential time savings observed in this study should be further evaluated using systematic cost-benefit analyses. In addition, subsequent research should concentrate on outcome-oriented end points, including the impact of continuous contactless monitoring on the early detection of clinical deterioration, the incidence of delirium, fall rates, and care-sensitive outcomes. The integration of activity tracking warrants further investigation. Finally, greater emphasis must be placed on the implementation of research findings in clinical care. A comprehensive understanding of how training concepts, technical support, interoperability with hospital information systems, and participatory implementation strategies influence long-term acceptance is imperative for translating technological feasibility into sustainable clinical practice [21,23].

### Conclusions

The outcomes of this study indicate that, from the perspective of nursing personnel, contactless monitoring may be both feasible and acceptable in selected inpatient settings. A comparison of geriatric and dialysis care reveals the necessity of implementing strategies tailored to local workflows and requirements. The findings from this study, which employed a mixed methods approach, suggest that the clinical context and specific application conditions influence how new technology is perceived and used. These findings underscore the importance of aligning implementation approaches with technical performance, clinical workflows, and nursing needs. In light of the exploratory nature of the study and the methodological limitations imposed on it, further research is necessary. This additional research should be conducted in larger and more diverse clinical settings. Future studies should validate these findings and investigate long-term usability, acceptance, and integration into routine care.

## Supplementary material

10.2196/93624 Multimedia Appendix 1 Guiding questions for the focus groups.

10.2196/93624 Multimedia Appendix 2 System usability after 4 weeks (T2), n=15.

10.2196/93624 Checklist 1 GRAMMS checklist.

## Abbreviations:

GRAMMS: Good Reporting of a Mixed Methods Study
ITU: intention to use
SUS: System Usability Score
TUI: Technology Usage Inventory
