# Supplementary material for: Radar-Based Contactless Monitoring in Different Inpatient Nursing Care Settings: Mixed Methods Feasibility Study
Source: JMIR Nurs. 2026 Aug 28;9:e93624. doi: 10.2196/93624 (PMC13524219; doi:10.2196/93624)
Supplement: Supplementary file 1 [file nursing-v9-e93624-s003.pdf]

**Appendix 1**

*Table 1 Guidelines for Good Reporting of A Mixed Methods Study (GRAMMS)*

| **Reporting Item** | **Where in Manuscript** |
|---|---|
| (1) Describe the justification for using a mixed methods approach to the research question | Methods/ Study design (p. 5) |
| (2) Describe the design in terms of the purpose, priority and sequence of methods | Aim (p. 4)<br>Methods/ Study design (p. 5), figure 1 |
| (3) Describe each method in terms of sampling, data collection and analysis | Sampling (p. 6)<br>Quantitative Data collection and analysis (p. 7)<br>Qualitative Data collection and analysis (p. 8) |
| (4) Describe where integration has occurred, how it has occurred and who has participated in it | Methods/ Study design (p. 5), figure 1 |
| (5) Describe any limitation of one method associated with the present of the other method | Strengths and Limitations (p. 19) |
| (6) Describe any insights gained from mixing or integrating methods | This design enabled the contextualisation and explanation of quantitative usability and acceptance data through the framework of qualitative insights. [Strengths and Limitations (p. 19)] |
| *GRAMMS - O'Cathain A, Murphy E, Nicholl J. The quality of mixed methods studies in health services research. J Health Serv Res Policy. 2008;13(2):92-98* | |
