# Supplementary material for: Radar-Based Contactless Monitoring in Different Inpatient Nursing Care Settings: Mixed Methods Feasibility Study
Source: JMIR Nurs. 2026 Aug 28;9:e93624. doi: 10.2196/93624 (PMC13524219; doi:10.2196/93624)
Supplement: Supplementary file 2 [file nursing-v9-e93624-s001.docx]

**Appendix 2**

Table 2 Guiding questions for the focus groups

- *What are your initial thoughts or experiences regarding the use of the Neteera system?*
- *What has been your experience of using the Neteera system so far?*
- *How would you rate the handling and integration of the system into your daily workflows?*
- *What advantages do you see in using the Neteera system for patient safety and quality of care?*
- *What role could the system play in nursing practice in the future?*
- *Does the system promote better care or does it lead to alienation of patients?*
- *What role does the setting play for you in assessing the support provided by Neteera?’*
- *What improvements would be necessary to optimally integrate the Neteera system into everyday care?*
- *What are your final thoughts or wishes for the future use of Neteera or similar monitoring systems in nursing care?*
